# Synthesis of Amines for Active Pharmaceutical Ingredients Using the Whole‐Cell Factory Saccharomyces Cerevisae

**DOI:** 10.1002/cbic.202500898

**Published:** 2026-05-10

**Authors:** Natalia Kwiatos, Stephen Ossel, Francesco G. Mutti

**Affiliations:** ^1^ Van ‘t Hoff Institute for Molecular Sciences HIMS‐Biocat University of Amsterdam Science Park 904 Amsterdam Netherlands

**Keywords:** alkaloids, biocatalysis, biocatalytic pathways, chiral amines, *Saccharomyces cerevisiae*

## Abstract

Whole‐cell biocatalysis offers a sustainable alternative to traditional chemical synthesis for producing pharmaceutically relevant, often chiral, amines and amino acids. *Saccharomyces cerevisiae* has emerged as a privileged microbial chassis due to its robustness, ease of genetic manipulation, and GRAS status. This concise review summarizes recent advances in metabolic and genetic engineering of *S. cerevisiae* for amine biocatalysis, focusing on strategies to overcome bottlenecks such as enzyme gene expression, cofactor regeneration, and precursor channeling. The first section covers state‐of‐the‐art methods for engineered strain construction, including genomic editing, optimization of gene expression (copy number, promoters, terminators, codon usage), and metabolic engineering (pathway balancing, compartmentalization, cofactor supply, transport proteins, auxiliary enzymes, and enzyme targeting via signal peptides), all enhancing product yields and enabling complex amine synthesis. The central section critically discusses compound families accessible via engineered *S. cerevisiae*, including various amines, amino alcohols, and amino acids such as l‐carnitine, ergothioneine, halogenated tryptamine, serotonin, psilocybin, spermidine, l‐ornithine, and mycosporine derivatives. Bioproduction of complex alkaloids, such as tropine derivatives (hyoscyamine and scopolamine) and ergot alkaloids, is also reviewed. Finally, current challenges and future perspectives are outlined, highlighting the integration of systems and synthetic biology tools to establish *S. cerevisiae* as a scalable platform for industrial amine production.

## Graphical abstract

This review highlights advances in engineering *Saccharomyces cerevisiae* for the sustainable biocatalytic production of pharmaceutically relevant amines, amino alcohols, amino acids, and complex alkaloids. It focuses on strategies to increase productivity, including enhanced enzyme expression, cofactor regeneration, precursor channeling, pathway balancing, and the removal of interfering enzymes, all aimed at enabling scalable industrial applications.

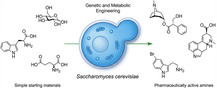

## 1 Introduction

Active pharmaceutical ingredients (APIs), which very often include amine functionalities, are used for the mitigation and treatment of diseases [1]. A significant portion of these are either directly sourced from, or derivatives of, compounds found in plants [2, 3]. These natural compounds possess a high degree of structural complexity and the challenges associated with their total synthesis have spawned an entire field of study to which a Nobel Prize was awarded [4, 5]. The efforts in the total synthesis of natural products have significantly advanced the development of modern organic chemistry. However, despite these advancements, the total synthesis of many valuable bioactive natural products remains commercially unviable due to low efficiencies. For example, (−)‐cyclopamine, a potential anticancer agent, (+)‐aberrarone, an antimalarial agent, as well as the antibiotics amycolamicin and darobactin A, are obtained with yields ranging from 1.4% to 5.7% on a one‐gram scale [6, 7, 8, 9].

Cultivation of plants remains a primary source for many of these compounds [10]. Due to the rising standards of living and prevalence of chronic ailments such as cancer and diabetes, the production of complex APIs through cultivation has grown increasingly insufficient. This leads to the need for the development of viable and industrially scalable routes for the synthesis of these compounds, which would also decrease the effect of environmental and geopolitical conditions on their global supply. Enzymatic or chemo‐enzymatic syntheses of these compounds from cheap starting materials, ideally sourced from renewable resources, are a promising alternative for traditional organic chemistry as enzymes offer high selectivity while using environmentally benign conditions and reagents [11, 12, 13, 14, 15, 16, 17, 18]. However, recombinant enzyme production can be complex and time consuming. Additionally, the use of enzymes introduces challenges such as biocatalyst isolation and, when required, purification, as well as cofactor dependency [19]. Whole‐cell biocatalytic systems (i.e., living, or resting but intact cells) may serve as an attractive alternative, as they not only offer a platform for enzyme gene expression but also mitigate issues related to enzyme immobilization and cofactor supplementation [20, 21, 22]. Whole‐cell in vivo biocatalysis consists of complete microorganisms engineered to perform a specific catalytic function [21]. These systems, several of which will be discussed in this mini‐review, represent alternatives to in vitro approaches (Table 1).

**TABLE 1:** Challenges and advantages of in vivo (growing or resting whole cells) and in vitro biocatalysis [21, 23].

	In vitro biocatalysis	In vivo biocatalysis
Advantages	Avoidance of side reactions. Fewer diffusion issues. Higher tolerance towards substrate, intermediate and product concentration.	Whole cells require no purification before use. Multistep cascade reactions with a single system possessing multiple catalytic activities. Endogenous cofactor regeneration.
Challenges	Separated production of enzymes. Immobilization needed for many cases. Lower stability.	Endogenous side reactions. Product separation from cells can be challenging. Substrate/product toxicity to living or resting cells. Difficult transport of substrates and products across the cell membranes or cell‐wall

Plant‐derived APIs can be produced both in their native plant sources and in engineered plant systems, including whole plants and plant cells or organ cultures [24, 25]. Plants better accommodate plant‐derived enzymes, can safely store metabolites that are toxic to microbes, and provide subcellular compartmentalization that supports complex pathway engineering, while photosynthesis enables low‐cost and sustainable synthesis [26]. However, these approaches face significant challenges, such as low and variable metabolite abundance, slow growth, complicated scale‐up, and sensitivity to environmental conditions. Field cultivation suffers from fluctuations in climate, pests, diseases, soil differences, and limited arable land, all of which introduce batch‐to‐batch variability and complicate consistent API composition and quality [27]. Even under controlled in vitro conditions, plant cell cultures often exhibit slow growth, genetic instability, and cell aggregation, and their cultivation requires technically demanding bioreactor systems [24]. These limitations have driven a major shift toward microbial and synthetic‐biology platforms—especially engineered yeast—which offer industrial familiarity, robust genetic tools, high‐density fermentation, and modular, high‐titer pathways for diverse small‐molecule APIs such as terpenoids, alkaloids, and phenylpropanoids [3].

*Escherichia coli* is the microorganism of choice for most industrial and academic applications [28]. This is largely due to its status as the genetically most studied microorganism, as well as the availability of a large variety of strains and tools for recombinant expression of genes encoding diverse enzymes. Despite these advantages, *E. coli* presents several limitations, including the absence of membrane‐bound organelles owing to its prokaryotic nature, lack of certain post‐translational modifications such as glycosylation, as well as its classification as a nongenerally recognized as safe (GRAS) organism [21]. In this context, *S. cerevisiae* has emerged as the workhorse eukaryotic microorganism for whole‐cell biocatalysis. Where *E. coli* falls short, *S. cerevisiae* can offer an alternative, as it is a GRAS organism and a eukaryote. Commonly known as baker's yeast, *S. cerevisiae* was used for brewing and baking even before the dawn of industrialized society. While at first being limited to simple fermentation processes, extensive efforts have allowed researchers to expand the scope of its application tremendously into the synthesis of pharmaceuticals and other high‐value chemicals [29]. Apart from its status as a GRAS organism, *S. cerevisiae* offers a number of advantages over other microorganisms as a platform for whole‐cell biocatalysis: excellent tolerance towards harsh reaction conditions, convenient genetic engineering and low cultivation cost [30]. Furthermore, the presence of inner membrane systems such as the endoplasmic reticulum (ER), where, for instance, most eukaryotic cytochrome P450 enzymes are localized, makes it especially suitable for the reconstruction of plant‐based biosynthetic cascades [30]. This has led to extensive work in recreating natural pathways for the synthesis of valuable compounds in yeast [31]. Despite these advantages, *S. cerevisiae* also comes with a set of challenges. Yeast is a significantly more complex organism than *E. coli* and this is reflected predominantly in the tightly regulated amino acid metabolism. This fact complicates the synthesis of amines with fermenting or resting whole cells, especially as this often involves amino acids as precursors.

There are other GRAS microbial cell factories that offer many advantages. Gram‐positive bacteria (e.g., lactic acid bacteria, *Corynebacterium glutamicum*) and other yeasts (e.g., *Kluyveromyces marxianus*, *Yarrowia lipolytica*) may grow faster, tolerate higher temperatures, secrete proteins more efficiently, or use broader carbon sources [31, 32, 33, 127]. *S. cerevisiae*, which is Crabtree‐positive, tends to overproduce ethanol on sugars, and shows limited native use of pentoses and mixed lignocellulosic substrates compared with nonconventional yeasts. *S. cerevisiae* also exhibits hyper‐glycosylation and relatively modest secretion, so alternative yeasts or filamentous fungi can outperform it for surface display or bulk extracellular enzyme production [34]. Thus, for thermophilic, mixed‐sugar, or high‐level extracellular enzyme processes, other GRAS systems can be technically or economically superior. However, *S. cerevisiae* has unmatched genetic tools, omics data, and regulatory familiarity, making it the default industrial yeast chassis [34].

This review aims to provide an overview of and critically analyze biocatalytic pathways for the synthesis of amines as pharmaceutically active compounds in *S. cerevisiae*. Potential strategies to improve efficiency of production strains will also be provided, specifically by discussing the combination of genomic editing and metabolic engineering [35].

## 2 Strain Construction

*S. cerevisiae* can be engineered to recombinantly produce enzymes or to express heterologous genes encoding enzymes for entire enzymatic cascades, either through integration of these genes into its genome or by introducing them on vectors.

Yeast vectors can be divided into a few categories [36, 37]. Yeast replicating plasmids (YRp) contain an autonomously replicating sequence (ARS) that allows independent replication; however, due to the absence of partitioning control, they are highly unstable and are frequently lost during cell division.

YCp vectors contain both an ARS and a centromere (CEN), resulting in chromosome‐like segregation during cell division. As a result, these types of vectors are stable but are maintained at low copy number [26, 27, 28]. The next type, episomal plasmids (YEps), are vectors based on the naturally occurring 2µ vector found in yeast [24, 30]. YEps can have up to 80 copies in a single cell [31]. Their stability depends on the presence of 2µ‐associated elements and host strain background, and their copy number is not tightly controlled. Yeast integrating plasmids (YIp) lack a yeast origin of replication and therefore must integrate into the host genome via homologous recombination, resulting in highly stable, single‐copy insertions [38].

Vector‐based expression is straightforward to perform—once the DNA is introduced into the host, the cells machinery expresses the new genes. This simplicity makes this method attractive for strain construction.

Yeast plasmids have been successfully applied in metabolic engineering but offer limited control over copy number and can suffer from segregational instability even under selective conditions [39]. In contrast, the high efficiency of homologous recombination in *S. cerevisiae* makes chromosomal integration a straightforward and reliable alternative, enabling stable insertion of defined numbers of genes. This precise control is particularly advantageous for the balanced and regulated expression of biosynthetic pathway genes [37, 40].

**FIGURE 1:**
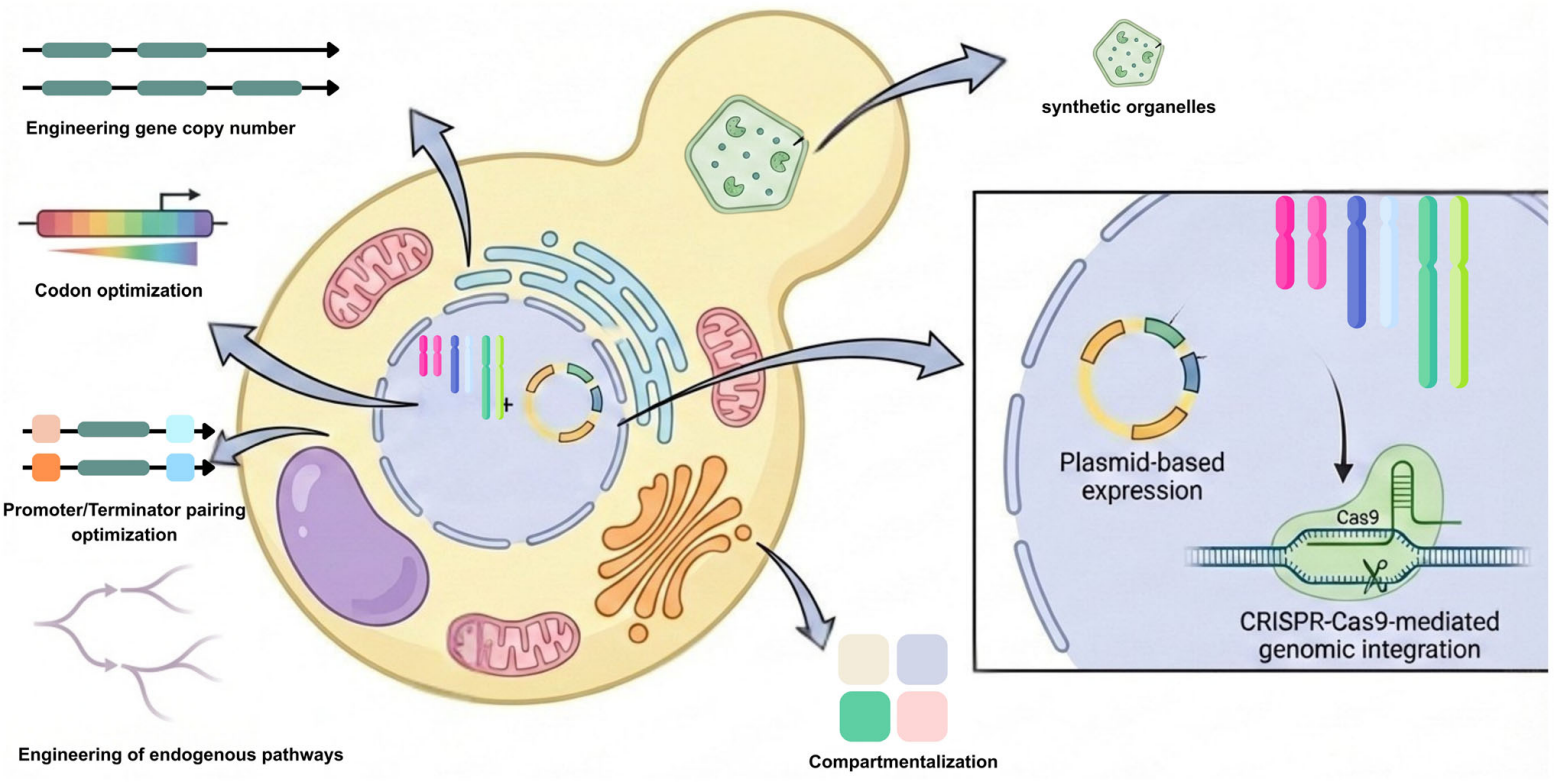
Visual overview of strategies for engineering *Saccharomyces cerevisiae* as a whole‐cell factory. Strain construction can be achieved via vector‐based expression or chromosomal integration, while gene expression can be optimized through copy number adjustment, promoter/terminator selection, and codon strength optimization. Metabolic engineering strategies include regulation of native and heterologous genes, pathway compartmentalization, genome editing, and engineering of membrane transporters to improve substrate uptake, intermediate trafficking, and product export.

Engineering *Saccharomyces cerevisiae* for biocatalysis involves multiple complementary strategies, including strain construction, precise genomic editing, optimization of gene expression, metabolic pathway engineering, and control over intracellular transport and compartmentalization, all of which collectively enable efficient and balanced heterologous enzyme gene expression and biosynthetic flux (Figure 1).

### 2.1 Genomic Editing

Genomic editing involves the alteration of the hosts chromosomal DNA. Vector‐based integration utilizes YIps, which contain targeting sequences homologous to sequences on the chromosome. Integration occurs via crossover recombination, a process native to the cell and can be used to introduce new genes, or to knock‐out existing genes [37]. The number of copies of the new gene integrated is dependent on the abundance of the target sequence and the number of YIps molecules introduced. The use of smaller YIps is usually desired as they are more easily prepared (for example easier amplification by PCR) and have a higher recombination efficiency [41].

Endonucleases are enzymes that recognize specific DNA sequences and create double‐stranded breaks (DSBs) [42]. These breaks are repaired by endogenous mechanisms, the process of which allows for gene integration, deletion, or alteration. In the past, systems such as zinc finger nucleases and transcription activator‐like effector nucleases, both known, respectively, as ZFN and TALEN, were used to introduce DSBs on specific locations [43, 44]. However, these methods suffer from low efficiencies and are time and labor intensive [45]. Thus, in recent years, they have largely been replaced by the more advanced CRISPR/Cas‐based systems, which offer high efficiency and precision [46]. Since its introduction to the field of genome engineering in 2013, CRISPR/Cas‐based systems have become the work‐horse of gene editing methods [47]. The system is based on the bacterial endonuclease Cas, which utilizes guide RNA (gRNA) to introduce DSBs on precise locations. Utilizing engineered gRNA targeting sequences, these cuts can be localized virtually anywhere on the genome. The number of tools developed using CRISPR‐Cas for *S. cerevisiae* is vast [48, 49, 50, 51]. A more comprehensive summary would far exceed the scope of this work. For this, the recent review by Wu et al. is recommended [46].

The choice of the integration site plays a role in the genomic stability of the inserted gene. Sites that are flanked by important genes are less prone to homologous recombination and thus more stable [52]. In addition to this, integration efficiency and expression strength also vary by genomic locus, making site selection a useful strategy for fine‐tuning gene expression [53]. Marker genes such as *HIS4*, *URA3*, and *LEU2* have been used as integration sites; however, these suffer from limited space [40]. This has led to the exploration of neutral sites, which are sites of whose modification does not affect hosts health. Among these, the most popular are δ‐sites, which are frequently occurring, making them attractive for multicopy integration of large biochemical pathways [54, 55]. Numerous sites are known and more are being discovered regularly (for example, X‐2, X‐3, X‐4, XI‐2, XI‐3, XI‐5, and XII‐5) . As such, research into their stability and expression efficiency is ongoing [30, 53].

In conclusion, genomic integration is superior to vector‐based expression with regard to genomic stability and expression control. Although many methodologies for genetic integration exist, the process remains more labor‐intensive than vector‐based expression. While genetic integration is generally preferred for production strains, pathway exploration or screening of enzyme activity in vivo is still best performed using vector‐based systems.

### 2.2 Optimizing Gene Expression

Vector copy number, integration site, as well as promoter and terminator choice are key strategies in fine‐tuning the expression levels of heterologous protein. This may be especially important in the expression of complex biochemical pathways, in which the activity of one or more enzymes may create a bottleneck. In this section, several strategies to increase gene expression are discussed.

#### 2.2.1 Improving Gene and Vector Copy Number

The most straightforward approach to enhancing expression levels is increasing the copy number of the target gene [56]. Multicopy vectors usually have a copy number up to 80; however, it has been demonstrated that this number can be increased even further. Destabilization of the vector‐based marker gene *URA3*—by fusing the protein to an ubi‐tag in addition to down‐regulating the gene by replacing the native *URA3* promoters with *HXT1*, *KEX2*, or *URA3‐d* promoters—led to a higher vector copy number [56]. In addition to this, deletion or overexpression of *RAF1*, a gene present in the native 2µ vector, also led to an increased copy number, although at the cost of stability [57]. It was also demonstrated that localization of the heterologous gene on the vector affects its copy number and stability, with integration downstream of *RAF1* or *REP1* on the native 2µ vector, leading to higher stability and expression levels. This was further increased by moving the native *TPI1* gene from the chromosome to the vector [58].

While chromosomally integrated genes are more stable than those on vectors, their expression strength is lower owing to their lower copy number [55]. Integration into common sites such as the δ‐site allows for incorporation of multiple copies of the same gene. Additionally, the nature of the site itself also influences expression strength of the gene, depending on the chromatin structure.

#### 2.2.2 Promoter and Terminator Engineering

Gene expression efficiency is greatly influenced by promoters and terminators [37]. Constitutive promoters (e.g., *TEF1*, *TDH3*, and *PGK1*) drive continuous expression and are associated with high expression levels [55, 59]. In addition to natural promoters, a range of synthetic promoters has been developed to further fine‐tune expression. The 2012 review by Da Silva and Srikrishnan offers a comprehensive overview [37]. Terminators influence mRNA stability and thus gene expression by increasing the rounds of translation that take place in the mRNAs lifespan. This makes them an important tool in the fine‐tuning of expression levels as well [60, 61]. Not unlike promoters, synthetic terminators have also been developed, some of them achieving 11 times the termination efficiency of natural terminators [62, 63].

#### 2.2.3 Codon Optimization

Traditionally, heterologous genes are optimized for the target host by replacing uncommon codons with more common ones, thereby increasing its codon adaptation index. For example, expression of green fluorescent proteins—a widely used tool in molecular biology—in *S. cerevisiae* led to 101‐fold increase fluorescence when the gene sequence was optimized for yeast [64].

Traditional codon optimization strategies treat all protein‐coding genes in a genome as equivalent and largely ignore cellular context. Consequently, they fail to capture key features of effective codon usage bias that can be critical for robust heterologous gene expression, and they also overlook evidence that neighboring codons coevolve. Newer optimization approaches address these limitations by incorporating cellular conditions and local codon dependencies, thereby improving the expression of heterologous enzymes in yeast [65, 66].

### 2.3 Metabolic Engineering

Metabolic engineering focuses on optimizing biosynthetic pathways. Beyond tuning heterologous gene expression through copy number, integration site, and promoter/terminator engineering, strategies include overexpressing or disrupting endogenous genes and modifying cofactor localization.

#### 2.3.1 Gene Regulation for Pathway Optimization

Overexpression or disruption of native genes is one of the most powerful strategies in metabolic engineering. Overexpression of native genes is like increasing the copy number of heterologous genes and may also include the use of stronger terminators or promoters. Deletion (knock‐out) of genes is especially useful for the disruption of metabolic or catabolic routes, giving control over the accumulation rate of specific compounds. A significant risk associated with gene knock‐out, however, is cell growth impairment. In these cases, downregulation of the relevant genes may be preferred over knock‐out. Downregulation can be achieved through exchanging native promoters and terminators to weaker variants [46]. To demonstrate the versatility of these techniques, Table 2 lists a number of examples.

**TABLE 2:** Examples of gene regulation for metabolic engineering.

Gene(s)	Operation	Method	Effect	Source
*scHEM1*	Overexpression	Increased gene copy number	Increased 5‐aminolevulinic acid production	[67]
*RKI1* and *TKL1*	Overexpression	Increased gene copy number	Increased erythrose‐4‐phosphate availability	[46]
*ARO7G141S* and *ARO4K229L*	Overexpression	Increased gene copy number	Promoted formation of aromatic amino acids	[68]
*FAD1*	Overexpression	Increased gene copy number	Increased FAD supply	[69, 70]
*βAS*, *ERG1*, *ERG9*, *ERG20*, *IDI* and *tHMG1*	Overexpression	Terminator engineering	Increased β‐amyrin supply	[62]
*SNZ3*, *RFC4* and *RPS18B*	Overexpression	Increased gene copy number	Increased SAM supply	[71]
*Pdi1*	Overexpression	Increased gene copy number	Increased production of disulfide bearing enzyme	[69]
*ZWF1*	Overexpression	Increased gene copy number	Increased NADPH supply	[30]
*GAPN*	Heterologous expression	Gene introduction	Increased NADPH supply	[30]
*ARG3*	Downregulation	Downregulation with weaker promoter	l‐ornithine accumulation	[72]
*PFK1* and *Pyk1*	Downregulation	Expression of dCas9 as expression regulator	Fructose‐6‐phosphate accumulation	[73]
*GCN4*	Upregulation	Deletion of *Tor1* and *Yih1* genes	Promotes the formation of amino acids.	[128]
*PFK26* and *PFK27*	Knock‐out	Gene deletion	Reduce glucose metabolism	[73]
*ARO10* and *PDC5*	Knock‐out	Gene deletion	Promotes formation aromatic amino acids	[74]

In *S. cerevisiae*, the shikimate pathway is a central metabolic route that converts phosphoenolpyruvate and erythrose‐4‐phosphate (E4P) into precursors for aromatic amino acids, while the Ehrlich pathway catabolizes amino acids to fusel alcohols and acids. Numerous target products, such as *p*‐coumaric acid and other plant‐like aromatics, use shikimate and chorismate derived metabolites or aromatic amino acids as precursors [39, 75]. Both pathways can be engineered to increase the yields of desired products. For instance, introducing feedback‐insensitive DAHP synthase and chorismate mutase (Aro4K229L, Aro7G141S) and increasing the availability of E4P enabled high‐level *p*‐coumaric acid production [74]. In another work, deleting *PDC1*, *PDC5*, *PDC6*, and *ARO10*, and substituting their activity with overexpression of a bacterial pyruvate decarboxylase, eliminated production of fusel alcohols and increased production of coumaric acid [39].

#### 2.3.2 Compartmentalization and Cofactor Supply

Compartmentalization is the spatial segregation of enzymes and metabolites within defined cellular or artificial compartments, such as mitochondria, peroxisomes, vacuoles, or synthetic nanocompartments, which create distinct microenvironments for biochemical reactions [76, 77, 78]. In *S. cerevisiae*, this strategy has been leveraged to enhance engineered metabolic cascades [79, 80]. Such an approach enables the enrichment of pathway enzymes and intermediates, the isolation of toxic proteins or metabolites from the cytosol, the minimization of flux loss to competing pathways, and the alignment of heterologous pathways with favorable local pools of precursors and cofactors. For example, relocating the entire heme synthesis pathway into yeast mitochondria and supplementing it with a more efficient bacterial‐derived pathway and folding chaperones can markedly increase heme production. Another example is the reconstruction of a complete agroclavine pathway from ergot fungi in *S. cerevisiae* by dividing the pathway into two submodules, one of which was anchored to the endoplasmatic reticulum (ER) to create a compartmentalized part of the cascade. Combining ER compartmentalization with enhanced NADPH supply and chromosomal integration ultimately led to agroclavine titers of 101.6 mg/L in flasks and 152.8 mg/L in fed‐batch fermentation, a 241‐fold improvement over the initial strain [79].

In yeast, compartmentalization is primarily achieved through: a) directing pathways to native organelles via signal peptides or targeting sequences; and b) constructing synthetic organelles or condensates—such as protein‐based nanocompartments, phase‐separated droplets, or optogenetically controlled clusters—to colocalize enzymes, enhance metabolic flux, and minimize side reactions [70, 78, 81, 82, 83].

In *Saccharomyces*, compartmentalization and cofactor supply are tightly interconnected: the choice of organelle for a biosynthetic pathway must align with local cofactor availability. Achieving high metabolic flux therefore often requires simultaneous optimization of both pathway localization and cofactor generation or transport. Cofactor availability can be enhanced through several approaches: a) expressing key cofactor‐generating enzymes in the same compartment as the synthetic cascade (typically the cytosol); b) relocating the entire pathway to an organelle with the high level of target cofactor, such as mitochondria for heme‐dependent or redox‐intensive reactions; and c) engineering cofactor regeneration systems [70, 80, 84].

#### 2.3.3 Transport Protein across Membranes

Transport of substrates, intermediates, products, and cofactors across biological membranes is widely recognized as a major bottleneck in the efficiency of artificial biosynthetic cascades in whole cells. In microbial cell factories, overall pathway performance depends not only on enzyme activities but also on how effectively small molecules move between the extracellular medium, cytosol, and specialized organelles such as peroxisomes and mitochondria [85, 86].

Organic molecules require membrane transporter proteins for efficient passage across lipid bilayers. Most native transport systems have evolved to handle endogenous metabolites, whereas many APIs and their intermediates may accumulate in inappropriate cellular compartments, become trapped in organelles, or be secreted only slowly because no compatible or high‐capacity transporter exists [85, 87]. Intracellular accumulation of reactive or hydrophobic molecules can damage membranes, disrupt energy metabolism, and activate stress responses, all of which reduce growth and product titers. These observations have motivated transporter engineering as a metabolic engineering strategy to enhance substrate import, prevent loss of intermediates, and promote efficient product and cofactor transport between organelles and out of the cell. For example, overexpression of the polyamine transporter gene *TPO1* increased spermidine production by enhancing product export and reducing its reuptake from the medium [88].

The *S. cerevisiae* genome encodes ≈340 membrane transporters, yet only a small fraction have been experimentally assigned substrates or functions, leaving much of the transportome uncharacterized [89, 90]. To address these knowledge gaps and support metabolic engineering efforts, several high‐throughput strategies have been developed, including transportome‐wide CRISPR disruption libraries coupled with biosensors to screen transporter effects on xenobiotic production, as well as isotope‐labeling–based assays for systematic profiling of native metabolite transporters [86, 89]. These and related screening platforms aim to identify transporters that limit substrate uptake, intermediate leakage, or product export in engineered yeast cell factories. In addition to exploiting native transport capacity, heterologous transporters can also be expressed in *S. cerevisiae* to enhance uptake or export of target molecules [91, 92, 93]. For instance, expression of plant transporters facilitated intracellular trafficking of intermediates in a reconstructed plant biosynthetic pathway for tropane alkaloid production [91].

## 3 Synthesis of Active Pharmaceutical Ingredients in S. cerevisiae

The strategies outlined above enable the rational engineering of *S. cerevisiae* strains capable of producing APIs from simple, renewable substrates at industrial scale. The practical implementation of these technologies is already underway, as illustrated by the recombinant production of protein hormone insulin for the treatment of diabetes [29]. However, other examples have been reported, including the antimalarial artemisinin, the anticancer compounds icariin and taxadiene, the antiviral shikimic acid, the antioxidant ergothioneine, and the dietary supplement l‐carnitine [73, 94, 95, 96, 97, 98].

### 3.1 Examples of Synthesis of Amines in S. cerevisiae

To offer insight into the types of amines produced in *S. cerevisiae* and the strategies utilized in the engineering of strains able to produce them, several cases will be presented. Table 3 gives a summary of all the covered examples in this review.

**TABLE 3:** Amine production in *S. cerevisiae*. Yields and scale are reported for each example presented.

Compound	Yield/Titer	Time/scale	Note	Source
(*R*)‐1‐phenylethylamine	50%, >99 e.e.	72 h/25 mL	From *rac*‐1‐phenylethylamine	[35, 99]
(*S*)‐2‐aminobutyric acid	0.6 mg/L	72 h/25 mL		[100]
(*S*)‐2‐aminobutanol	0.42 mg/L	72 h/2.5 mL		[100]
l‐carnitine	20 mg/L	24 h/10 mL		[98]
l‐ergothioneine	600 mg/L	75 h/1 L	Fed‐batch	[73]
Halogenated tryptophan	0.1–3.6 mg/L	24 h/0.5 mL	Mono‐ and di‐halogenated with Br and Cl	[101]
Halogenated tryptamine	0.05–2.4 mg/L	24 h/0.5 mL	Mono‐ and di‐halogenated with Br and Cl	[101]
4‐aminobutyric acid	10.2 g/L	96 h/100 mL		[102]
Spermidine	224 mg/L	24 h/1 L	Fed‐batch	[88]
l‐ornithine	778 mg/L and 5.1 g/L	50 mL and 3 L	Shake‐flask and fed‐batch, respectively	[72]
N‐methylpyrrolinium	18 mg/L	5 mL		[103]
Tropine	0.13 mg/L and 6 mg/L	120 h/10 mL and 48 h/0.3 mL		[104, 105]
Pseudotropine	0.08 mg/L	120 h/10 mL		[104]
Hyoscyamine	30 µg/L	120 h/10 mL		[91]
Scopolamine	30 µg/L	120 h/10 mL		[91]
Chanoclavine‐I	0.8 mg/L	72 h/25 mL		[70]
Cycloclavine	529 mg/L	160 h/1 L	Fed‐batch	[69]
Festuclavine	86 mg/L	160 h/1L	Fed‐batch	[69]
d‐lysergic acid	1.4 mg/L	50 h/4 L	Fed‐batch	[106]

### 3.2 Enantiomeric Enrichment

**SCHEME 1:**
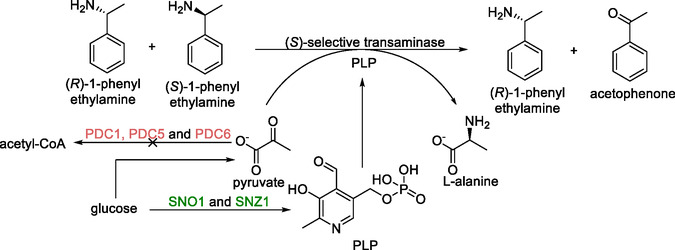
Kinetic resolution of *rac*‐1‐phenylethylamine. Glucose is used as the sole carbon source for the biosynthesis of amine‐acceptor pyruvate and cofactor PLP. The genes depicted in red were deleted to engineer the pyruvate accumulating strain. Genes that were upregulated (by cultivation in thiamine‐deficient conditions) are shown in green. Genes presented in the scheme: *PDC1*, *PDC5*, *PDC6—*genes encoding pyruvate decarboxylases, *SNO1* and *SNZ1*—genes encoding enzymes involved in pyridoxine metabolism.

Enantiomeric enrichment can be achieved by removing one enantiomer from a racemic mixture (also referred to as kinetic resolution). This strategy is often used to produce chiral amines and utilizes enzymes as they offer excellent enantioselectivity. To test the viability of this process in *S. cerevisiae*, a model reaction for the enantiomeric enrichment of *rac*‐1‐phenylethylamine was set up (Scheme 1) [99].

This reaction was catalyzed by transaminases. To identify a suitable candidate, a series of enzymes from different species were separately integrated into the genome and screened for activity. From this pool, a ω‐transaminase from *Chromobacterium violaceum* was found to be the best performing enzyme, giving 45% conversion and 83% *e.e.* after 200 h. Interestingly, it was also found that concentrations above 15 mM of *rac*‐1‐phenylethylamine resulted in slower cell growth, although, during fermentation, 25 mM of *rac*‐1‐phenylethylamine was nonlethal to the cells.

To improve conversion and enantiomeric purity, several issues were addressed [35]. First, the supply of pyruvate as the amine acceptor for the transamination reaction, was investigated (Scheme 1). To do this, the ω‐transaminase encoding gene from *C. violaceum* was expressed from a vector in a previously developed strain. This strain contained a series of deletions of pyruvate decarboxylase coding genes (*PDC1*, *PDC5*, and *PDC6*), resulting in accumulation of pyruvate. Indeed, conversion increased by 5% although this strain required C_2_ supplementation for growth and was therefore not investigated further. Next, increasing the concentration of pyruvate in the growth medium was attempted, but this approach did not result in any increased conversion. This was most likely due to the effect of elevated extracellular pyruvate levels on intracellular pH.

The availability of the cofactor PLP was addressed by supplementation of the medium with differing concentrations of the compound. As this was not successful, another method to increase intracellular PLP was investigated, namely cultivation in thiamine deficient medium. It was hypothesized that this would increase the production of intracellular PLP, as thiamine suppresses genes involved in its biosynthesis. This, in combination with the expression of additional copies of the transaminase coding gene, resulted in 50% conversion with > 99% *e.e.* after 72 h. It was proposed that the fermentation time could be reduced further by the conversion of acetophenone to 1‐phenylethanol with reductases. This approach would prevent product inhibition of the transaminases, improving enzymatic activity. However, this option was not explored.

### 3.3 (*S*)‐2‐Aminobutyric Acid and (*S*)‐2‐Aminobutanol

**SCHEME 2:**
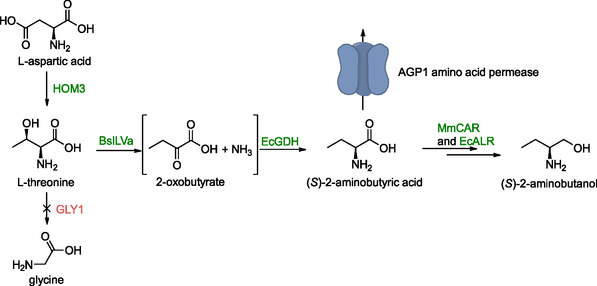
Pathway from l‐threonine to (*S*)‐2‐aminobutyric acid and (*S*)‐2‐aminobutanol. In the first step, both the endogenous threonine deaminase encoded by *ILV1* and a heterologous threonine deaminase were active. Vector expressed inhibition‐less *HOM3* (green) resulted in a 1.5‐fold increase of l‐threonine levels. Deletion of *GLY1* (red) to avoid consumption of l‐threonine for the formation of glycine had no effect. The expression of the permease encoded by *AGP1* was induced by elevated levels of l‐threonine. Genes presented in the scheme: BsILVa—threonine deaminase (a PLP‐dependent ammonia lyase), EcGDH—glutamate dehydrogenase, HOM3—aspartate kinase, GLY1—threonine aldolase, MmCAR—carboxylic acid reductase, and EcALR—aldehyde reductase.

(*S*)‐2‐aminobutyric acid is a chiral building block for the synthesis of pharmaceutically active amines. Its synthesis in *S. cerevisiae* starting from l‐threonine, was achieved with two different sets of enzymes. In the first, a threonine deaminase (*BsILVa*) from *Bacillus subtilis* and a mutated glutamate dehydrogenase (*EcGDH*) from *E. coli* were expressed from a vector (Scheme 2) [100, 107]. The second strategy involved upregulation of a native threonine deaminase encoding gene (*ILV1*) by replacing its native promoter with the stronger *GDP1* promoter in combination with vector‐based expression of two copies of a glutamate dehydrogenase encoding genes from *Solanum lycopersicum*. Both strategies combined resulted in a titer of 0.4 mg/L (*S*)‐2‐aminobutyric acid. However, the latter also yielded an elevated 2‐ketobutyric acid concentration (1.6 mg/L). Accumulation of this compound was most likely the result of feedback inhibition on glutamate dehydrogenase.

To compensate for this, the supply of l‐threonine was increased, which led to a higher total (*S*)‐2‐aminobutyric acid concentration albeit decreased the conversion yield. The increased intracellular concentration of l‐threonine was thought to promote the expression of genes encoding enzymes that compete with threonine deaminase. Surprisingly, while the conversion yield decreased, the fraction of (*S*)‐2‐aminobutyric acid excreted by the cells increased. The overexpression of the *AGP1* gene, encoding an amino acid permease, which facilitates (*S*)‐2‐aminobutyric acid export and is induced by l‐threonine, was thought to account for this observation. Although this transporter could relieve feedback inhibition of glutamate dehydrogenase, its influence was likely outweighed by the upregulation of competing enzymes described earlier.

Metabolic engineering was also attempted to increase l‐threonine availability. A feedback‐insensitive variant of the *HOM3*‐encoded aspartate kinase, a key enzyme in l‐threonine biosynthesis, was expressed from a 2μ vector, leading to a 150% increase in (*S*)‐2‐aminobutyric acid yield. Deletion of a gene (*GLY1*) coding for an enzyme that irreversibly converts l‐threonine to glycine did not have any effect on the titer and resulted in impaired cell growth.

In this (*S*)‐2‐aminobutyric acid‐producing strain, genes for a carboxylic acid reductase *from Mycobacterium marinum*, a phosphopantetheinyl‐dependent transferase from *Bacillus subtilis* and an aldehyde reductase (*EcALR*) from *E. coli* were expressed, resulting in the formation of (*S*)‐2‐aminobutanol (Scheme 2). Genomic integration resulted in a 3‐fold higher (*S*)‐2‐aminobutanol titer, which after pH optimization to 7, reached 0.42 mg/L.

### 3.4 l‐Carnitine

**SCHEME 3:**
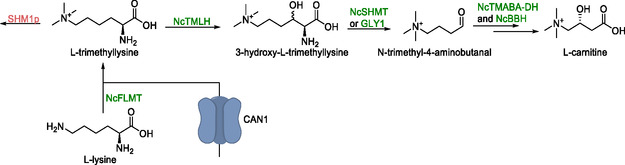
Metabolic pathway for the conversion of l‐trimethyllysine to l‐carnitine. The deletion of *SHM1p* (red) led to an increase in l‐carnitine production. The transport protein encoded by *CAN1* was responsible for up‐take of extracellular l‐trimethyllysine. Genes presented in the scheme: *SHM1p—*yeast serine hydroxymethyltransferases, *NcTMLH*—l‐trimethyllysine hydroxylase, *NcSHMT*—serine hydroxymethyl‐transferase, *GLY1p*—yeast threonine aldolase, *NcTMABA‐DH*—trimethylaminobutyraldehyde dehydrogenase, *NcBBH*—butyrobetaine hydroxylase, and *NcFLMT*—free l‐lysine methyltransferase.

l‐Carnitine, which is used as a dietary supplement, is a derivative of l‐lysine. The de novo synthesis of l‐carnitine was achieved in *S. cerevisiae* (Scheme 3) [98]. From *Neurospora crassa*, a series of genes encoding the enzymes l‐trimethyllysine hydroxylase (TMLH), serine hydroxymethyl‐transferase (SHMT), trimethylaminobutyraldehyde dehydrogenase (TMABA‐DH), and butyrobetaine hydroxylase (BBH) were expressed in *S. cerevisiae*. In nature, a serine hydroxymethyl‐transferase (SHMT) typically converts serine to glycine via elimination of formaldehyde, which is accepted by tetrahydrofolate to form 5,10‐methylene‐tetrahydrofolate. However, SHMT was previously found to promiscuously catalyze the retro‐aldol cleavage of 3‐hydroxy‐l‐trimethillysine, thereby behaving as a 3‐hydroxy‐l‐trimethillysine aldolase. Because *S. cerevisiae* was also suspected to possess native 3‐hydroxy‐l‐trimethillysine aldolase activity, the SHMT‐encoding gene from *Neurospora crassa* was expressed from vector, while the remaining enzymes were integrated onto the genome. This allowed testing of l‐carnitine production with and without heterologously expressed SHMT. l‐carnitine was formed in both cases, but conversion was 25% higher in the presence of heterologous SHMT. The gene responsible for the native reactivity toward 3‐hydroxy‐l‐trimethillysine in *S. cerevisiae* was later identified as *Gly1*, encoding a ‘true’ aldolase.

To avoid dependency on external supply of l‐trimethyllysine, a free l‐lysine methyltransferase (FLMT)‐encoding gene was expressed. However, this modification did not increase in the l‐carnitine production rate. In fact, no l‐carnitine formation was observed in strains expressing the FLMT gene, even in the presence of l‐trimethyllysine. In attempts to enhance l‐trimethyllysine availability, *CAN1* was identified as the permease mediating its uptake from the extracellular environment. However, overexpression of this gene to increase intracellular l‐trimethyllysine concentrations was not attempted.

To further improve the l‐carnitine yield, multiple genes encoding enzymes competing with l‐trimethyllysine hydroxylase were deleted. Deletion of *SHM1p* resulted in a 9‐fold increase in conversion to l‐carnitine; however, this also caused severe growth impairment. The best conversion of l‐trimethyllysine to l‐carnitine was achieved using all four genes from *N. crassa*, reaching 0.4% conversion of extracellular l‐trimethyllysine (20 mg/L) on a 10 mL scale.

### 3.5 Ergothioneine

**SCHEME 4:**
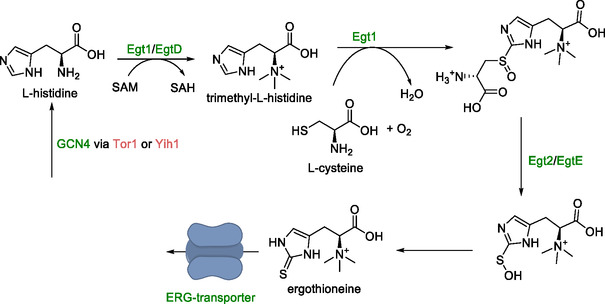
Fungal pathway for the conversion of l‐histidine and l‐cysteine to ergothioneine. Upregulation of *GCN4* (in green) by deletion of *Tor1* and *Yih1* (in red) did not result in an increase in ergothioneine titers. Neither did the expression of an ergothioneine (ERG) transporter encoding gene. Genes presented in the scheme: *Egt1* and *EgtD—*SAM dependent methyltransferases, *Egt2* and *EgtE*—hercynylcystein sulfoxide lyase, *Tor1*, *Yih1*, and *GCN4*—genes involved in nitrogen metabolism.

Ergothioneine is a dietary supplement with antioxidant properties, which is currently primarily extracted from natural sources. Its synthesis in *S. cerevisiae* has been achieved utilizing l‐histidine and l‐cysteine (Scheme 4) [73]. For this, genes encoding a SAM‐dependent methyltransferase (*Egt1*) from *N. crassa* and a hercynylcystein sulfoxide lyase (*Egt2*) from *Claviceps purpurea* were expressed. Additional copies of both genes only increased conversion to ergothioneine by 25%. This prompted the exploration of bottlenecks, including feedback inhibition by removing ergothioneine from the cell. However, the introduction of a gene coding for an ergothioneine transporter did not yield any improvement. Additional studies suggested that the observed effect resulted from the subcellular localization of these transporters, which were primarily associated with the vacuolar membrane instead of the plasma membrane.

Upregulation of the *GCN4* gene, which plays a role in the supply of amino acids, through deletion of the *Tor1* and *Yih1* genes did not result in an increased conversion to ergothioneine. Finally, growth medium optimization was performed. Initial attempts to increase the concentrations of relative amino acids, namely l‐cysteine, l‐methionine, and l‐histidine from 0.1 to 2 g/L resulted in cell death. Screening of the concentration of many components yielded 76 mg/L for l‐arginine, l‐histidine and l‐methionine, and 0.4 mg/L for pyridoxine as optimal supplementation. On a 1 L scale, this increased ergothioneine titers from 30 to 600 mg/L.

### 3.6 Halogenated Tryptophan and Tryptamine

**SCHEME 5:**
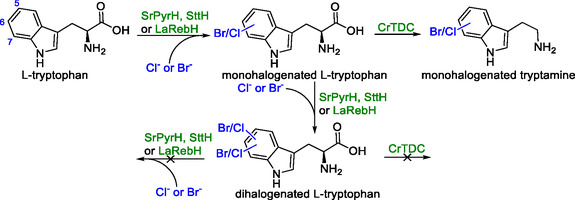
In blue: positions 5, 6, and 7 on tryptophan. Mono‐ and dihalogenation with both bromide and chloride was observed, while trihalogenation was not. The tryptophan decarboxylase CrTDC showed catalytic activity towards halogenated tryptophan, allowing for the formation of monohalogenated tryptamine, while dihalogenated tryptamine formation was not observed. Genes presented in the scheme: SrPyrH—5‐halogenase, SttH—6‐halogenase, LaRebH—7‐halogenase, and *CrTDC*—tryptophan decarboxylase.

Halogenated indole moieties are common motives in APIs owing to the beneficial properties induced by the presence of the halide [108]. Moreover, halogenated indoles serve as important building blocks for a wide scope of compounds [109]. In spite of this, their synthesis remains challenging, requiring toxic reagents that offer only limited regioselectivity [110]. To overcome these issues, the synthesis of halogenated tryptophan and tryptamine has been explored in yeast [3, 101]. To this end, three tryptophan halogenases encoding genes, *SrPyrH* (5‐selective) from *Streptomyces rugosporus*, *SttH* (6‐selective) from *Streptomyces toxytricini* and *LaRebH* (7‐selective) from *Lentzea aerocolonigenes*, were expressed under control of the *TDH3* promoter. To address potential supply problems with FADH_2_, a partner flavin reductase (*LaRebF*) from *L. aerocolonigenes* was also integrated. In the presence of *LaRebF* gene and an appropriate halide source, all three halogenases showed successful bromination and chlorination of tryptophan, with titers between 0.1 and 3.6 mg/L on a 0.5 mL scale. The coexpression of genes encoding halogenases with different regioselectivity led to the formation of dihalogenated tryptophans (5,6‐; 5,7‐; and 6,7‐); however, no trihalogenated molecules were observed (Scheme 5). Expression of a tryptophan decarboxylase (*CrTDC*) coding gene under the control of the *TEF1* promoter gave halogenated tryptamine titers between 0.05–2.4 mg/L. However, dihalogenated tryptamine was never observed.

### 3.7 Serotonin

**SCHEME 6:**
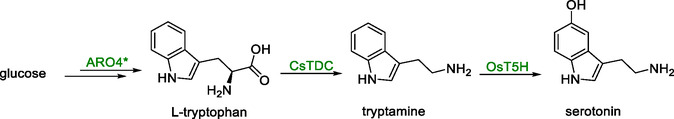
Metabolic pathway for the conversion of glucose to serotonin. A feedback insensitive mutant of an enzyme involved in the production of l‐tryptophan was used to increase the availability of this amino acid (encoded by ARO4* gene). A tryptophan decarboxylase (CsTDC) and a tryptamine hydroxylase (OsT5H) were used to convert tryptophan to serotonin.

Serotonin is a tryptophan‐derived indoleamine that functions as a key neurotransmitter in mammals, playing essential roles in regulating both the nervous and immune systems [111]. At present, serotonin is mainly produced either through total chemical synthesis or via chemical conversion of 5‐hydroxytryptophan, which is typically extracted from the seeds of *Griffonia simplicifolia*. However, these routes rely on costly precursors and generate considerable chemical waste. Consequently, biotechnological production of serotonin has emerged as a promising and more sustainable alternative. Recent work has demonstrated a promising and sustainable strategy for serotonin biosynthesis in *S. cerevisiae.* Two heterologous genes were introduced: tryptophan decarboxylase from *Clostridium sporogenes* (*CsTDC*) and tryptamine 5‐hydroxylase from *Oryza sativa* (*OsT5H*), enabling the stepwise conversion of tryptophan to serotonin (Scheme 6). Random integration of these genes into *Ty4* loci generated multiple strains with varying gene copy numbers, from which the best producer was selected. To further enhance precursor availability, the *ARO4* gene—encoding a key regulator of the shikimate pathway leading to l‐tryptophan biosynthesis—was replaced with a feedback‐resistant variant (ARO4*) and integrated into the same genomic sites. Combined with cultivation medium optimization, these modifications redirected metabolic flux toward serotonin formation, achieving titers of 250 mg/L in a 1 L bioreactor.

### 3.8 Psilocybin

Recent advances in metabolic engineering have enabled *S. cerevisiae* to synthesize psilocybin, the psychoactive compound naturally produced by *Psilocybe* species and currently under clinical investigation for the treatment of depression, anxiety, and cluster headaches [112].

**SCHEME 7:**
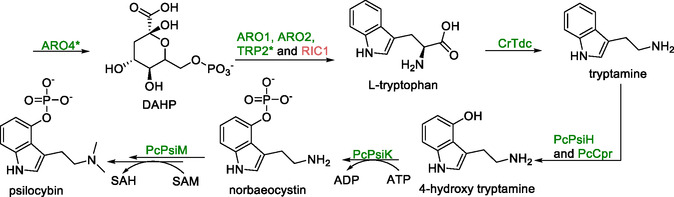
Metabolic pathway for the conversion of l‐tryptophan to psilocybin. Availability of l‐tryptophan was increased by overexpression of ARO1 and ARO2 and deletion of regulatory gene RIC1. Expression of feedback insensitive mutants ARO4* and TRP2* were ineffective in increasing the availability of l‐tryptophan. Other genes presented in the scheme: CrTDC—tryptophan decarboxylase, PcPsiH—monooxygenase, PcCpr—cytochrome P450 reductase, PcPsiK—kinase, and PcPsiM—methyltransferase.

The biosynthetic pathway from *Psilocybe cubensis* was successfully reconstructed in yeast by heterologous expression of genes coding for four key enzymes: tryptophan decarboxylase (*CrTdc*), monooxygenase (*PcPsiH*), kinase (*PcPsiK*), and methyltransferase (*PcPsiM*) (Scheme 7). These genes were integrated into defined chromosomal loci using CRISPR/Cas9‐mediated genome editing to ensure stable, balanced expression under strong constitutive promoters. To enhance monooxygenase activity, a cytochrome P450 reductase from *P. cubensis* (*PcCpr*) was coexpressed, leading to a substantial increase in psilocybin titers. Additional metabolic optimizations targeted the tryptophan biosynthetic pathway, the main precursor supply route. Overexpression of *ARO1* and *ARO2* genes, which encoded enzymes that convert 3‐deoxy‐d‐arabino‐heptulosonate‐7‐phosphate (DAHP) to chorismate, together with deletion of *RIC1*, a regulator of aromatic amino acid metabolism, further boosted psilocybin levels. Interestingly, the expression of feedback‐insensitive mutants of *ARO4* and *TRP2* unexpectedly reduced titers, emphasizing the need for careful balancing of precursor flux. Through stepwise pathway and host optimization, psilocybin production reached 627 ± 140 mg/L in controlled fed‐batch fermentations using minimal synthetic medium.

### 3.9 4‐Aminobutyric Acid

**SCHEME 8:**
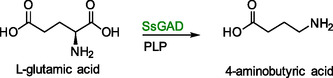
Conversion of l‐glutamic acid to 2‐aminobutyric acid. *SsGAD*—PLP‐dependent glutamate decarboxylase.

4‐aminobutyric acid is a nonproteinogenic amino acid with antihypertensive and antiepileptic properties. Additionally, it is a valuable intermediate in the production of more complex pharmaceuticals. Its synthesis in *S. cerevisiae* was achieved through vector‐based expression of a gene coding for a PLP‐dependent glutamate decarboxylase from *Streptomyces* sp. (Scheme 8) [102].

In this case, optimizing the concentrations of PLP and l‐glutamic acid in the medium was sufficient to obtain an overall productivity of 62.6 g/L, accumulated over 10 consecutive batch cycles. More specifically, the first cycle yielded a titer of 10.2 g/L 4‐aminobutyric acid, which gradually decreased to 1.8 g/L by cycle 10. Therefore, recycling of the engineered *S. cerevisiae* cells was not pursued beyond cycle 10.

Higher concentrations of either component lowered conversion rates, most likely due to cellular toxicity. By centrifuging the fermentation mixture and decanting the supernatant, the cells could be reused up as mentioned above. The loss of activity upon subsequent recycling was attributed to thermal deactivation during biotransformation and to repeated centrifugation from one batch to the next. Previous studies indicated that PLP production could be increased through cultivation in thiamine deficient media. This would enhance the cost‐efficiency and practical applicability of the method by eliminating the need for PLP cofactor supplementation [35]. Additionally, metabolic engineering through upregulation of l‐glutamic acid producing genes might make supplementation of l‐glutamic acid unnecessary. However, both strategies were not employed in this study.

### 3.10 Spermidine

**SCHEME 9:**
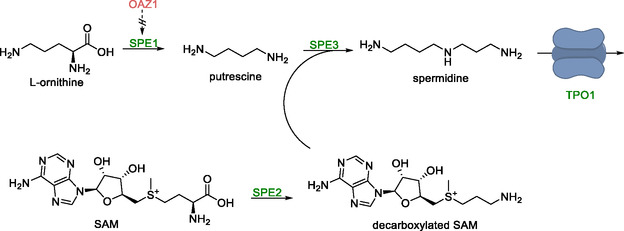
Conversion of l‐ornithine and SAM to spermidine using ornithine decarboxylase (encoded by *SPE1*), S‐adenosylmethionine decarboxylase (*SPE2*) and spermidine synthase *SPE3*. Spermidine export in the engineered strain was mediated by the transporter TPO1. The deletion of the *OAZ1* gene enhanced ornithine decarboxylase activity.

Spermidine is a polyamine derived from putrescine which is studied for the treatment of type 2 diabetes and skin aging. Low levels of this compound are naturally formed in wild‐type *S. cerevisiae*. The enzymes ornithine decarboxylase converts l‐ornithine into putrescine, which then reacts with a decarboxylated derivative of SAM via spermidine synthase to form spermidine [88]. The decarboxylation of SAM is in turn facilitated by S‐adenosylmethionine decarboxylase (Scheme 9).

Vector‐based overexpression of all three genes under the control of the GPD promoter, combined with deletion of the *OAZ1* gene that inhibits *SPE1*, resulted in a titer of 0.61 mg/L. The yield was improved by 191% by overexpression of *TPO1*, a gene encoding a polyamine transport protein. Removal of spermidine from the cell lowered its intracellular concentration, possibly preventing feedback inhibition of *SPE3*, thereby increasing enzymatic activity. Optimization of *TPO1* transport activity was achieved by increasing the gene copy number and adjusting the medium pH to 2.4. This, in combination with decreased aeration during fermentation that was supposed to upregulate enzymes involved in precursor synthesis, increased the spermidine titer to 63.6 mg/L on a 50 mL scale.

Notably, spermidine accumulation was observed only after glucose depletion. Glucose induced inhibition of a gene (*FMS1*) encoding a key enzyme in the spermidine metabolism, was thought to be responsible for this effect. To overcome this, the pathway was expressed in the xylose‐fermenting *S. cerevisiae* strain SR8. Fermentation in xylose and limited glucose increased the spermidine titer to 224 mg/L resulting in the most efficient spermidine producing strain of *S. cerevisiae*.

### 3.11 l‐Ornithine

**SCHEME 10:**
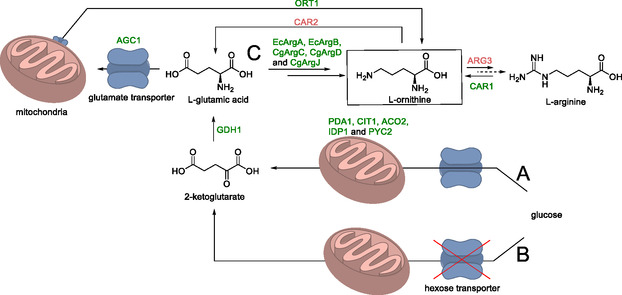
Overview of the synthesis of l‐ornithine from glucose via l‐glutamic acid. Starting from glucose: (A) overexpression of Krebs cycle enzyme genes or (B) downregulation of a hexose transporter resulted in increased 2‐ketoglutarate availability. Overexpression of *GHD1*, *AGC1*, and *ORT1* augmented native l‐ornithine synthesis by facilitating l‐glutamic acid and l‐ornithine transport between the cytosol and mitochondria, and increased l‐glutamic acid supply. (C) Heterologous enzymes ARGA, ARGB, ARGC, ARGD, and ARGJ formed an additional route to l‐ornithine localized in the cytostol. Deletion and downregulation of *CAR2* and *ARG3*, together with overexpression of *CAR1*, facilitated l‐ornithine accumulation by preventing conversion to l‐glutamic acid and l‐arginine. Other genes presented in the scheme: EcArgA—N‐acetyltransferase, EcArgB—acetylglutamate kinase, CgArgC—N‐acetyl‐γ‐glutamyl‐phosphate‐reductase, CgArgD—acetylornithine aminotransferase, and CgArgJ—ornithine acetyltransferase. Some enzymes involved in the pathway for the conversion of l‐ornithine to l‐arginine are omitted for simplicity.

l‐Ornithine is used as a dietary supplement to support liver function and serves as a biosynthetic precursor for various complex natural products. It occurs as an intermediate in the synthesis of l‐arginine and the increase of its intracellular levels has been the subject of extensive metabolic engineering (Scheme 10) [72].

Because of the complex nature of the amino acid production chain in *S. cerevisiae* and the role of l‐ornithine as an intermediate herein, a number of strategies to increase its production were possible. Three will be discussed here. First, conversion of l‐ornithine to l‐arginine via ornithine carbamoyltransferase (*ARG3*) was attenuated by replacing the native promoter of *ARG3* with weaker promoters (*HXT1* or *KEX2*), which substantially increased l‐ornithine titers while avoiding strict l‐arginine auxotrophy. It was attributed to reduced l‐arginine levels, which likely led to both the overexpression of genes coding for enzymes in the downstream metabolism and the alleviation of their feedback inhibition. Subsequently, *CAR2*, a gene encoding an enzyme involved in the conversion of l‐ornithine back to l‐glutamic acid, was deleted yielding a further 10% increase in the l‐ornithine titer.

**SCHEME 11:**
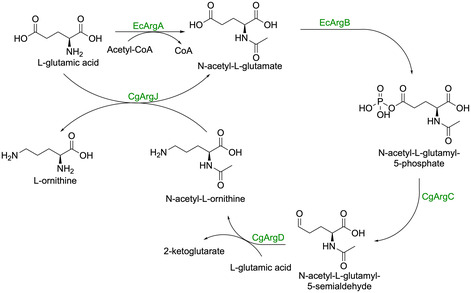
The biocatalytic cycle for the synthesis of l‐ornithine from l‐glutamic acid.

Second, native l‐ornithine synthesis was boosted by improving mitochondrial/cytosolic trafficking (*ORT1*, *AGC1*) and glutamate supply (*GDH1*), and by expressing a heterologous cytosolic acetylated‐derivative cycle (*Ec ArgA/ArgB* and *Cg ArgC/ArgD/ArgJ*), which together strongly increased l‐ornithine production (Scheme 11).

The final strategy involved increasing the availability of the l‐ornithine precursor 2‐ketoglutarate. This was achieved in two distinct ways. The first focused on improving the productivity of the central carbon‐metabolism through overexpression of genes encoding enzymes that are part of the Krebs cycle. To this end, *PDA1*, *CIT1*, *ACO2*, *IDP1*, and *PYC2* were overexpressed using episomal vectors. The result of this was a 28% increase in the l‐ornithine titer. This strain was then used in a 3 L fed‐batch experiment with limited glucose availability and aeration, giving a titer of 5.1 g/L. The reason for this sharp increase is the Crabtree effect as explained below.

At high glucose concentrations, the carbon metabolism in *S. cerevisiae* bypasses the Krebs cycle and is redirected towards anaerobic fermentation to ethanol, even in the presence of oxygen. Because the Krebs cycle is responsible for a significant portion of the supply of compounds such as NADH and 2‐ketoglutarate, bypassing it significantly reduces the availability of these species. In the fed‐batch experiment the glucose concentration was kept low, avoiding the Crabtree effect and thus increasing the l‐ornithine titer significantly.

An alternative method to circumvent the Crabtree effect was also explored, namely the downregulation of a gene encoding a hexose transporter protein involved in glucose uptake. In addition, manipulation of the arginase reaction (*CAR1*), which converts l‐arginine to l‐ornithine and urea, was considered as part of the l‐ornithine degradation module. Summing up, the integration of genetic and process‐level optimization was the decisive factor in reaching 5.1 g L^−1^, the highest reported titer throughout this study.

### 3.12 N‐Methylpyrrolinium and Tropine

**SCHEME 12:**
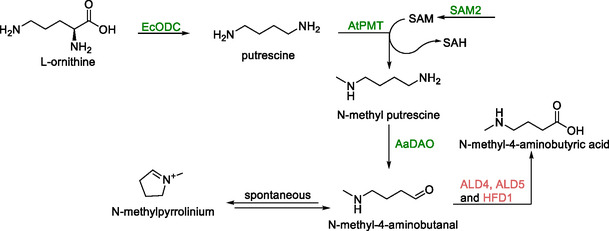
The synthesis of N‐methylpyrrolinium from l‐ornithine. Supply of SAM for AtPMT was increased through overexpression of *SAM2*. Conversion of N‐methyl‐4‐aminobutanal to N‐methy‐4‐aminobutyric acid was prevented by deletion of the *ALD4*, *ALD5* and *HFD1* genes. AtPMT—putrescine N‐methyltransferase, AaDAO—methylputrescine oxidase, ALD4, ALD5, and HFD1—aldehyde dehydrogenases.

N‐methylpyrrolinium is an important building block for the synthesis of pharmaceutically active amines. Its production from l‐ornithine in *S. cerevisiae* was achieved by constructing a strain expressing genes encoding ornithine decarboxylase (*EcODC*) from *Erythroxylum coca* and putrescine N‐methyltransferase (*AtPMT*) from *Anisodus tanguticus* from a vector, in combination with genomic expression of a gene encoding methylputrescine oxidase (MPO, coded for by *AaDAO*) from *A. tanguticus* (Scheme 12) [103]. This strain yielded 2 mg/L N‐methylpyrrolinium after 24 h. Deletion of a series of aldehyde dehydrogenase coding genes (*ALD4*, *ALD5*, and *HFD1*) increased the strains productivity by 370% by lowering degradation of N‐methyl‐4‐aminobutanal. Hereafter, supply of SAM was improved by overexpression of *SAM2*, resulting in a further 230% increase in the N‐methylpyrrolinium titer, bringing it to a final 18 mg/L.

**SCHEME 13:**
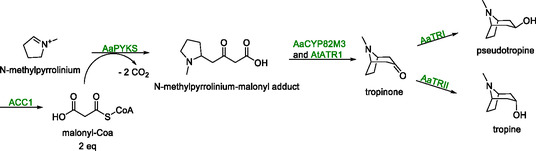
The enzymes encoded by *AaPYKS*, *AaCYP82M3*/*AtATR1* and *AaTRI*/*AaTRII* catalyze the conversion of N‐methylpyrrolinium to tropine and pseudo‐tropine. Overexpression of the native *ACC1* gene increased the yield through enhancing the supply of malonyl‐Coa. Notably, two equivalents of malonyl‐CoA are required to form the N‐methypyrrolinium‐malonyl adduct.

Tropine alkaloids are N‐heterocyclic compounds used in the treatment of neuromuscular conditions, such as Parkinson's disease or nerve agent poisoning. The production of tropine, and derivatives thereof, in *S. cerevisiae* has been achieved using two different methods [104, 105]. The first reported synthesis of tropine in *S. cerevisiae* was developed by the same group that constructed the N‐methylpyrrolinium producing strain discussed in Scheme 12. Genomic expression of genes encoding a pyrrolidine ketide synthase (*AaPYKS*) and a tropinone synthase (*AaCYP82M3*) from *Anisodus acutangulus* in combination with a cytochrome P450 reductase from *A. thaliana* (*AtATR1*) resulted in a strain capable of forming tropinone (Scheme 13) [104]. However, this strain accumulated N‐methylpyrrolinium, indicating incomplete conversion. This was addressed by overexpression of the native *ACC1* gene, which is involved in the production of malonyl‐CoA, on which the N‐methylpyrrolinium consuming PYKS was dependent. Integration of either of two tropinone reductase‐encoding genes (*AaTRI* or *AaTRII*) from *A. thaliana*, yielded 0.13 mg/L tropine and 0.08 mg/L pseudotropine after 120 h on a 10 mL scale.

**SCHEME 14:**
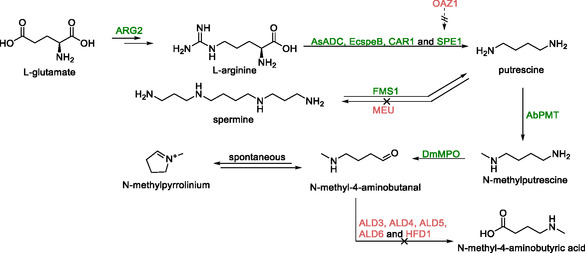
Synthesis of N‐methylpyrrolinium from l‐glutamic acid. The removal of several aldehyde dehydrogenase genes (bottom, red) avoids conversion of N‐methyl‐4‐aminobutanal to N‐methyl‐4‐aminobutyric acid. Furthermore, deletion of *OAZ1*, a *SPE1* inhibitor, and *MEU*, a gene coding for an enzyme involved in the conversion of putrescine to spermidine increased the flux to the desired product. Other genes presented in the scheme: EcspeB—agmatine ureohydrolase, AsADC—arginine decarboxylase, DmMPO—methylputrescine oxidase, and AbPMT—putrescine methyltransferase.

The second study focused on constructing a platform for the synthesis of N‐methylpyrrolinium (Scheme 14) [105]. Rather than starting solely from l‐ornithine, the researchers choose to start from l‐glutamic acid, increasing the number of routes to N‐methylpyrrolinium. Vector based expression of gene encoding arginine decarboxylase (*AsADC*) from *Avena sativa* and an agmatine ureohydrolase (*EcspeB*) from *E. coli* introduced a new pathway to produce putrescine. Simultaneous vector‐based overexpression of *SPE1* and genomic overexpression of *CAR1*, *SPE1*, and *FMS1*, augmented the native pathway. Removal of the *MEU* gene, which codes for an enzyme that plays a role in the irreversible conversion of putrescine to spermine, and the *OAZ1* gene, which codes for an *SPE1* inhibitor, created a strain that produced 86 mg/L putrescine after 48 h on a 2 mL scale.

Integration of genes encoding putrescine methyltransferase from *Atropa belladonna* and a truncated methylputrescine oxidase from *Datura metel* resulted in the production of N‐methylpyrrolinium. Removal of a series of aldehyde dehydrogenase coding genes (*ALD3*, *ALD4*, *ALD5*, *ALD6*, and *HFD1*) to prevent conversion of N‐methyl‐4‐aminobutanal to N‐methyl‐4‐aminobutyric acid resulted in a strain producing 40 mg/L N‐methylpyrrolinium after 96 h. The notably higher N‐methylpyrrolinium titer of this strain compared to the previously discussed one, might be explained by the expanded putrescine supply, alternative methylputrescine oxidase (MPO) and putrescine N‐methyltransferase (PMT) enzymes used, and the deletion of an additional aldehyde dehydrogenase.

**SCHEME 15:**
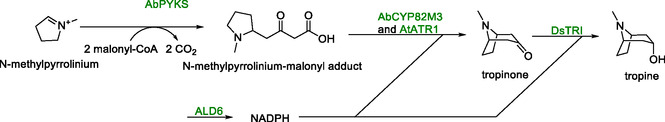
Conversion of N‐methylpyrrolinium to tropine using *AbPYKS* (pyrrolidine ketide synthase), *AbCYP82M3*/*AtATR1* (tropinone synthase/cytochrome P450 reductase) and *DsTRI* (tropinone reductase). *ALD6* (aldehyde dehydrogenase) was previously deleted but reinstated as its activity resulted in increased NADPH supply, which benefited the activity of the enzymes in the cascade.

Expression of genes encoding a pyrrolidine ketide synthase (*AbPYKS*) and a tropinone synthase (*AbCYP82M3*) from *Atropa belladonna*, together with a cytochrome P450 reductase from *A. thaliana* (*AtATR1*) and a tropinone reductase (*DsTRI*) from *Datura stramonium* resulted in tropine producing strain (Scheme 15). It was found that reintroduction of the previously removed *ALD6* gene increased the tropine titer to 1.5 mg/L. An increase in NADPH supply from the aldehyde dehydrogenase encoded by *ALD6* was thought to positively affect the activity of the NADPH dependent ATR1 and TR1, thus explaining this observation. Introduction of an additional copy of *AbPMT*, and two new copies of *DsPMT* from *D. stramonium* further increased the yield to 3.3 mg/L. Further media optimization eventually led to 6 mg/L tropine concentration after 48 h.

### 3.13 Hyoscyamine and Scopolamine

**SCHEME 16:**
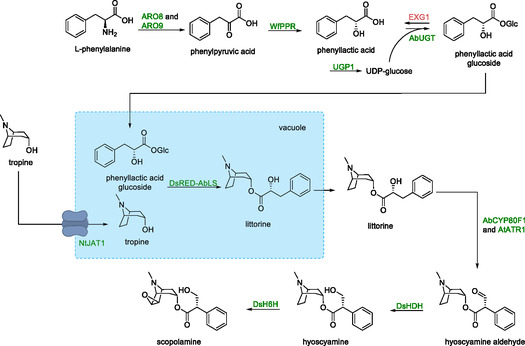
Top: Conversion of l‐phenylalanine to phenyllactic acid glucoside by overexpressing *ARO8*, *ARO9* and *UGP1* (its gene products catalyze the isomerization of glucose‐6‐phosphate to glucose‐1‐phosphate and conversion of glucose‐1‐phosphate to UDP‐glucose) together with the heterologous *WfPPR* (coding for phenylpyruvate reductase) and AbUGT (phenyllactic acid UDP glycosyltransferase). EXG1 (glucosidase gene) was deleted to lower degradation of phenyllactic acid glucoside back to phenyllactic acid. In blue box: synthesis of littorine by a *Discosoma* sp. red fluorescent protein fused to the N‐terminus of *A. belladonna* littorine synthase (encoded by *DsRED‐AbLS*) in the vacuole together with increased tropine flux to the vacuole by the AT1 alkaloid transporter (*NtJAT1*). Bottom right: Conversion of littorine to hyoscyamine and scopolamine by *AbCYP80F1*/*AtATR1*, *DsHDH* (hyoscyamine dehydrogenase) and *DsH6H* (oxygenase).

The tropine derivatives hyoscyamine and scopolamine were synthesized in *S. cerevisiae* (Scheme 16) [91].

Overexpression of two native genes encoding aromatic aminotransferases (*ARO8* and *ARO9*) was used to increase flux to phenylpyruvic acid. Genes encoding phenylpyruvate reductase from *Wickerhamia fluorescens* and phenyllactic acid UDP glycosyltransferase from *A. belladonna* were then integrated into the genome resulting in the formation of phenyllactic acid glucoside, a crucial intermediary in the synthesis of both hyoscyamine and scopolamine.

Overexpression of *UGP1* and deletion of *EXG1* served to augment this biosynthetic pathway by improving coreagent availability and preventing phenyllactic acid glucoside degradation, respectively.

In plants, the synthesis of the tropine alkaloid intermediate littorine is performed by littorine synthase inside the vacuole. Expression of a gene from *Atropa belladonna* coding for a littorine synthase (*AbLS*) hindered yeast growth and yielded no measurable activity, suggesting that the enzyme was stalled in the secretory pathway. The N‐terminus of the enzyme, which in plants directs the protein to the vacuole, was not compatible with *S. cerevisiae*. Hence, the enzyme was fused to red fluorescent protein from *Discosoma* sp. (*DsRED*), which led to functional expression and normal cell growth. To increase tropine availability for littorine synthesis in the vacuole, a gene encoding the alkaloid transporter (*NtJAT1*) from *Nicotiana tabacum* was expressed.

Here‐after genes encoding for oxygenase (*DsH6H*), hyoscyamine dehydrogenase (*DsHDH*) from *D. stramonium*, and *A. belladonna* littorine mutase (*AbCYP80F1*) were integrated into the genome. Consequently, hyoscyamine and scopolamine were produced at titers of 10.2 µg/L and 1 µg/L, respectively. Additional copies of *WfPPR* and *DsH6H* were integrated, while the native *LEU2* gene, which codes for a NADH recycling 3‐isopropylmalate dehydrogenase, was also overexpressed. Together with media optimization through supplementation with 2‐oxoglutarate and Fe^2+^, both of which increase the activity of the enzyme encoded by *DsH6H*, this resulted in titers of 30 µg/L for both hyoscyamine and scopolamine. The modest yields observed may reflect the extended length of the biosynthetic pathway combined with the relatively low activities of the heterologous plant enzymes.

### 3.14 Ergot Alkaloids

**SCHEME 17:**
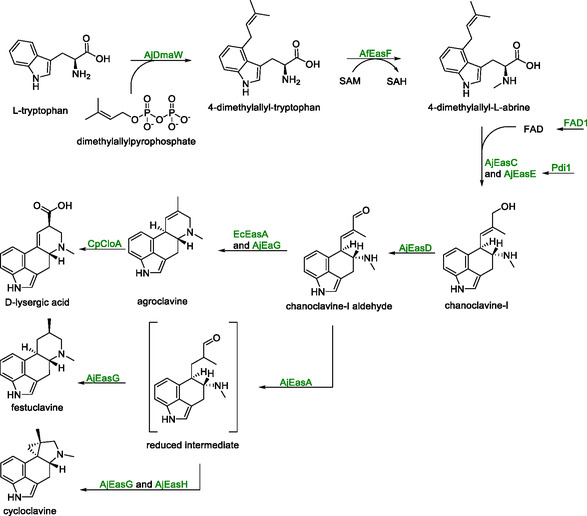
Conversion of l‐tryptophan to the common intermediate chanoclavine‐I aldehyde with enzymes encoded by the *AjDmaW* (a prenyl transferase), *AfEasF* (a methyltransferase), *AjEasC* (an oxidase), *AjEasE* (a flavin‐dependent oxidoreductase) and *AjEasD* (a dehydrogenase) genes. Expression of *AjEasA* (an ene‐reductase), *AjEasG* (an imine reductase) and *AjEasH* (an oxidase) led to the formation of festuclavine and cycloclavine while expression of *EcEasA* (a redox isomerase), *AjEaG* (an imine reductase) and *CpCloA* (a heme‐dependent oxygenase and oxidase) led to the formation of d‐lysergic acid.

Ergot alkaloids are a family of compounds, which are used in the treatment of neurological conditions such as Parkinson's disease and dementia. Current production involves the cultivation of crops that are infected with fungi, e.g., *Claviceps purpurea*. Submerged fermentation of engineered *C. purpurea* strains is also employed; however, both methods are limited by the diversity of ergot alkaloids produced, complicating downstream processing [106]. To address this, *S. cerevisiae* has been genetically engineered to produce specific ergot alkaloids. All biosynthetic pathways for this purpose start from l‐tryptophan and diverge at the common intermediate chanoclavine‐I (Scheme 17) [70].

Genomic integration of genes encoding a prenyl transferase (*AjDmaW*) from *Aspergillus japonicus* and a methyltransferase (*AfEasF*) from *Aspergillus fumigatus* led to the formation of 4‐dimethylallyl‐l‐abrine. Subsequent integration of two genes from *A. japonicus* (*AjEasC* and *AjEasE*) led to detectable amounts of chanoclavine‐I [70]. Both enzymes encoded by the two genes possessed N‐terminal targeting sequences, directing AjEasC to the peroxisome and AjEasE to the secretory pathway, respectively.

To determine the impact of these sequences on enzymatic activity, variations of each were examined. For *AjEasC*, removal of the peroxisomal targeting sequence led to slightly elevated chanoclavine‐I production. In contrast, altering the targeting sequence of *AjEasE* completely abolished chanoclavine‐I production, indicating that the original *A. japonicus* signal sequence is essential for its functional expression. To this point, titers of chanoclavine‐I were 0.4 mg/L after 72 h on a 25 mL scale. Overexpression of the native genes *FAD1*, for increased FAD supply, and *Pdi1*, for improved folding of *AjEasE*, increased the yields by 50% and 33%, respectively.

Genomic integration of *easD*, *easA*, *easG*, and *easH* (in addition to the earlier cluster genes) resulted in yeast strains producing both festuclavine and cycloclavine, with the addition of *easH* shifting the major product to cycloclavine [69]. To address the low titers of chanoclavine‐I, an extensive screening of gene copy number on its titer was performed. One additional copy of *AjEasG*, *Pdi1* and *FAD1*, two additional copies of *AjDmaW* and *AjEasD* and three additional copies of *AjEasC* gave a strain that yielded a 529 mg/L cycloclavine and 86 mg/L festuclavine after 160 h of fed‐batch fermentation on a 1 L scale.

Integration of an alternative set of genes, *EcEasA* from *Epichloë coenophiala*, *AjEasG* from *A. japonicus* and *CpCloA* from *Claviceps purpurea*, led to the formation of d‐lysergic acid [106]. Notably, the *EcEasA* used in these examples differed from that used for the synthesis of cycloclavine. While the earlier version encoded a reductase, an isomerase variant was used for the synthesis of d‐lysergic acid. For this strain, only additional copies for *FAD1*, *Pdi1*, *AfEasF*, *AjEasC*, and *AjEasD* were integrated. Additionally, a stronger promoter was used for *AjEasE*. This strain yielded 1.4 mg/L d‐lysergic acid after 50 h in a 4 L fed‐batch experiment. The substantial difference in titer between cycloclavine and d‐lysergic acid was not addressed, but may result from limited catalytic efficiency of the CpCloA and EcEasA enzymes.

Further optimization for ergot alkaloid producing strains might involve improving the availability of the cofactors SAM and NADPH. Previous examples achieved this through overexpression of native genes such as *SAM2* and *ZWF1*, respectively (see Table 2 and Scheme 12).

### 3.15 Mycosporine‐Like Amino Acids

Mycosporine‐like amino acids are adducts with antioxidant and UV‐protective properties. These compounds are naturally synthesized by marine organisms and cyanobacteria. To evaluate the potential for their production on an industrial‐scale, *S. cerevisiae* strains capable of producing shinorine and porphyra‐334 were engineered [113].

**SCHEME 18:**
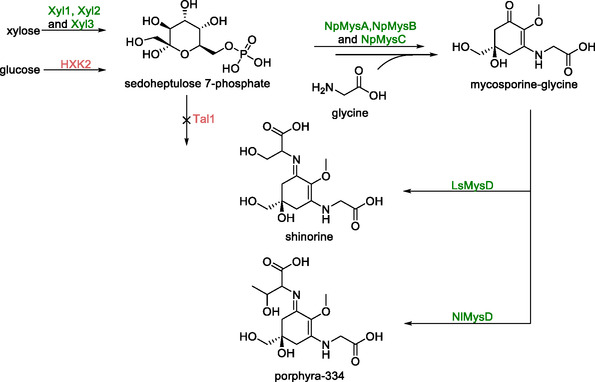
Pathway from xylose and glucose utilizing enzymes coded by *NpMysA*, *NpMysB*, *NpMysC*, and *LsMysD* from *Lynbya* sp. (for shinorine) and *NlMysD* from *N. Linckia* (for porphyra‐334). Optimization of this pathway was performed by introducing a set of xylose utilizing enzymes from *S. stipites* and removing the native genes *HXK2* and *Tal1*.

Both shinorine and porphyra‐334 are formed from the common intermediate mycosporine‐glycine (Scheme 18). To achieve this, genes encoding a 2‐demethyl‐4‐deoxogadusol synthase (*NpMysA*), a methyltransferase (*NpMysB*) and an ATP‐grasp enzyme (*NpMysC*) from *Nostoc punctiforme* were randomly integrated on the numerous δ‐sites of the genome. Screening of transformants with differing copy numbers of these genes led to the identification of an optimal strain carrying 5 copies of *NpMysA* and 4 copies of both *NpMysB* and *NpMysC*. Furthermore, to increase the availability of the mycosporine‐glycine precursor sedoheptulose 7‐phosphate (S7P), the native *HXK2* gene was deleted, and a series of genes for xylose metabolizing enzymes from *Scheffersomyces stipites* (*Xyl1*, *Xyl2*, and *Xyl3*) were integrated into the genome. Finally, the native gene *Tal1*, which codes for a transaldolase, was deleted to increase the S7P pool. d‐Ala‐d‐Ala ligase encoding genes, were then individually integrated into the mycosporine‐glycine producing strain, resulting in the formation of shinorine (for *LsMysD* from *Lyngbya* sp.) and porphyre‐334 (for *NlMysD* from *Nostoc linckia*) with titers of 1.53 g/L and 1.21 g/L respectively in 5 L fed‐batch fermentation after 120 h.

## 4 Summary and Outlook

This review showcased the current utilization of *S. cerevisiae* as a host organism for amine production, even though this microorganism has naturally been evolved for amino acid metabolism rather than amine production. Through a combination of heterologous gene expression for enzymes, cofactor balancing, precursor supply optimization, and transporter engineering, a wide range of valuable compounds has been biosynthesized, including chiral amines (via kinetic resolution), nonproteinogenic amino acids (e.g., (*S*)‐2‐aminobutyric acid, 4‐aminobutyric acid, and l‐ornithine), polyamines (e.g., spermidine, N‐methylpyrrolinium), N‐heterocycles and alkaloids (e.g., tropine, tropine derivatives, and ergot alkaloids), nutraceuticals and cosmeceuticals (e.g., ergothioneine, l‐carnitine, and mycosporine‐like amino acids), and halogenated indole derivatives (e.g., halotryptophans, halotryptamines). Yields vary considerably—from microgram to gram per liter scale—depending on pathway complexity, enzyme compatibility, precursor availability, and the degree of host metabolic rewiring.

Notable high‐yield examples highlight the capacity of *S. cerevisiae* to serve as a production host for amines and related compounds. Expression of a heterologous glutamate decarboxylase encoding gene enabled the synthesis of 4‐aminobutyric acid (GABA) at up to 62.6 g/L, representing one of the highest reported amine concentrations in yeast [102]. Another successful example is engineering of the mycosporine‐like amino acid pathway, which resulted in titers exceeding 1 g/L in 5 L fed‐batch fermentations, which shows the potential for cosmeceutical production at industrially relevant scales [113]. l‐ornithine accumulation reached 5.1 g/L in fed‐batch cultivation through suppression of the Crabtree effect [72]. This is a perfect example of how central carbon metabolism can be redirected to favor precursor supply. In contrast, more complex pathways leading to plant alkaloids or halogenated derivatives generally yielded in the milligram to microgram per liter range, illustrating the challenge of balancing precursor supply, cofactor regeneration, and heterologous enzyme activity in these biosynthetic cascades.

Cofactor availability is frequently pointed to as a critical bottleneck in the biosynthesis of amines and related nitrogen‐containing compounds. Many of the enzymes employed in these pathways depend on cofactors such as PLP, NADPH, FAD, or SAM, and insufficient intracellular pools often constrain productivity. Several strategies were explored to overcome these limitations. For example, cultivation under thiamine‐deficient conditions was shown to increase intracellular PLP levels, thereby enhancing the activity of PLP‐dependent enzymes [99]. Similarly, overexpression of *SAM2*, encoding an isoform of SAM synthetase, improved titers of SAM‐requiring products by expanding the available methyl donor pool [103]. To address redox imbalance, regeneration of NADPH through overexpression of *ZWF1* (glucose‐6‐phosphate dehydrogenase) proved beneficial for pathways relying on NADPH‐dependent reductases [30].

Another critical factor in amine production is the intracellular metabolic flux, which is strongly influenced by the availability of key precursors such as l‐threonine, l‐ornithine, l‐glutamate, or pyruvate. For instance, simultaneous enhancement of native and heterologous l‐ornithine biosynthetic pathways in *S. cerevisiae* resulted in a 426% increase in l‐ornithine titers [72]. Similarly, removal of feedback inhibition in threonine biosynthesis, combined with precursor supplementation, significantly improved (*S*)‐2‐aminobutyric acid production [100]. Nevertheless, attempts to increase precursor pools are often constrained by native regulatory mechanisms and competing pathways that divert flux away from the target product.

Targeted engineering of nitrogen metabolism has also been applied to expand precursor pools. For example, downregulation of an enzyme in the l‐arginine biosynthetic pathway led to accumulation of upstream intermediates, which was successfully exploited to increase l‐ornithine production [72]. Such interventions illustrate how regulatory circuits of amino acid metabolism can be rewired to support amine biosynthesis.

Transport engineering is another key strategy to improve amine production, as efficient import and export of metabolites can relieve intracellular inhibition and enhance titers. For example, overexpression of *AGP1*, an amino acid permease, increased excretion of (*S*)‐2‐aminobutyric acid, helping to circumvent feedback inhibition [100]. Similarly, enhanced expression of the polyamine transporter coding gene *TPO1* significantly raised spermidine titers by exporting the product and reducing intracellular accumulation [88].

In addition to plasma membrane transport, intracellular trafficking of metabolites is an underexplored but potentially important factor. For example, directing the movement of precursors such as l‐glutamate from mitochondria or enabling substrate availability for vacuolar enzymes could further improve pathway efficiency [72, 91]. Intracellular compartmentalization adds another layer of complexity to amine biosynthesis in yeast, and targeted engineering of metabolite routing within the cell may offer new opportunities for optimization.

Gene copy number and expression context significantly influence pathway performance. The level of enzyme production is highly dependent on whether the corresponding genes are integrated into the genome or carried on in vectors. While vectors often allow higher expression rates, they suffer from low genetic stability, making genomic integration the preferred option for stable biocatalytic strains. Nevertheless, plasmid expression remains a useful strategy for rapid enzyme testing prior to integration. Gene copy number and integration site are also crucial parameters for expression strength. For example, Kim et al. applied CRISPR/Cas9‐mediated integration of three mycosporine‐like amino acid biosynthetic genes into multiple genomic θ‐sites, generating strains with varying gene dosage and integration patterns [113]. Screening revealed that the optimal combination of gene copy number and genomic location produced the highest activity. More broadly, multicopy genomic integration of pathway genes often leads to higher titers compared with plasmid‐based expression, likely due to more stable expression and reduced metabolic burden. In addition, careful balancing of enzyme levels within a pathway can tune product distribution, as demonstrated in ergot alkaloid synthesis, where adjusting the ratio of *EasG* to *EasH* determined the relative formation of cycloclavine versus festuclavine [106].

Despite impressive progress in heterologous amine production, many pathways remain at the proof‐of‐principle stage, with titers in the milligram‐per‐liter range. Achieving industrially relevant yields will require systematic, multilevel optimization of pathways and host metabolism. Systems‐level approaches, including multiplex genome editing, dynamic pathway regulation, and modular expression systems, offer opportunities to overcome feedback inhibition, pathway imbalances, and metabolic bottlenecks.

Cofactor and redox balance will remain central to further improvements. Engineering robust regeneration systems for NADPH, PLP, and SAM is likely to enhance the efficiency of many pathways, particularly those involving reductases or methyltransferases. Transporter engineering represents another frontier: proper localization and tuning of exporters and importers can alleviate intracellular product inhibition and enable continuous or high‐density fermentation.

Alternative carbon sources may also expand production potential. For example, xylose‐fermenting strains have enabled higher spermidine titers by avoiding glucose repression and the Crabtree effect [88] suggesting that nonglucose substrates could unlock further improvements across other pathways.

Finally, the development of generic ‘amine chassis strains’ of *S. cerevisiae* could provide a versatile foundation for diverse amine production. Such strains, with preoptimized precursor pools, cofactor balance, and minimized byproduct pathways, would streamline engineering of new biosynthetic routes and facilitate rapid translation of laboratory successes into industrial applications.

In conclusion, *S. cerevisiae* remains an underexplored and underutilized microorganism for the synthesis of high‐value, structurally complex amines with important biological activities. However, the knowledge gained from the metabolic and enzyme engineering efforts described in this review is expected to guide the development of further engineered strains with improved production efficiency and broader structural diversity of products. In this context, the introduction of new‐to‐nature biocatalytic pathways—designed and validated in vitro and incorporating engineered enzymes—into *S. cerevisiae* may provide solutions to several of the challenges described in this review [114, 115, 116]. Such pathways could decouple amine production from native metabolism, thereby reducing metabolic interference and enhancing productivity. Although the toxicity of novel intermediates along the pathway may present additional challenges, it has been demonstrated that microorganisms can be evolved to acquire increased tolerance to toxic compounds [117]. Altogether, these advances highlight *S. cerevisiae* as a highly promising and versatile platform organism, poised to play a central role in the sustainable biotechnological production of high‐value amines in the near future. In this context, the Design–Build–Test–Learn (DBTL) cycle has emerged as a powerful framework for developing *S. cerevisiae* as a microbial cell factory. New designs are proposed, strains are constructed, performance is evaluated, and the resulting data guide subsequent rounds, making strain engineering more systematic and efficient [118]. In yeast, the Design step is strongly supported by genome‐scale metabolic models (GEMs) such as Yeast9, which capture known metabolic reactions and gene–enzyme relationships to predict fluxes, growth, and byproduct formation [119]. Enzyme‐constrained GEMs further incorporate protein allocation, improving predictions under industrially relevant conditions; for example, ecYeast8 enabled the design of a strain producing 70‐fold higher intracellular heme [120, 121]. During the Build phase, in silico designs are realized by assembling DNA parts and integrating them into the genome or plasmids. Standardized toolkits (e.g., YeastFab, YTK) and automated platforms facilitate construction of large combinatorial libraries of pathway variants. CRISPR/Cas‐based multiplex editing allows simultaneous modification of multiple loci, accelerating exploration of gene combinations and pathway configurations, and has been applied extensively in yeast metabolic engineering. Tools such as CasEMBLR enable one‐step assembly and integration of multiple DNA parts into targeted genomic sites, supporting complex pathway insertions and combinatorial constructs in vivo while improving throughput [46, 122, 123]. In the Test step, strain performance is quantified by measuring growth, product titer, yield, and omics profiles, often in high‐throughput formats. Emerging technologies are making this phase increasingly dynamic: genetically encoded biosensors can link intracellular metabolite levels to fluorescence or reporter outputs, allowing rapid screening of large libraries and real‐time feedback on pathway activity. Dynamic control elements, including inducible or feedback‐regulated promoters and metabolite‐responsive circuits, enable conditional flux control, mitigate toxic intermediate accumulation, and improve overall performance. Such strategies are critical for multistep pathways, where static overexpression often leads to flux imbalances [124].

Finally, in the Learn step, Test data are analyzed using statistics, GEMs, or machine learning to identify key design features and guide the next DBTL cycle. ML‐guided DBTL approaches in *S. cerevisiae* have increased titers of products such as *p*‐coumaric acid and β‐carotene while evaluating only a small fraction of possible designs [125, 126].

Biosynthetic cascades in *S. cerevisiae* are moving toward AI‐assisted, dynamically controlled, and spatially organized cell factories, supported by advanced genome editing and, increasingly, multispecies design strategies. Consequently, amine production in yeast is expected to follow this trajectory, evolving into intelligent, modular, and spatially engineered whole‐cell platforms capable of efficiently integrating cofactor balance, precursor supply, enzyme compartmentalization, and dynamic regulation.

## Funding

This work was supported by HORIZON EUROPE Marie Sklodowska‐Curie Actions (Grant 101153173).

## Conflicts of Interest

The authors declare no conflicts of interest.
